# Seismic Evidence for a Geosuture between the Yangtze and Cathaysia Blocks, South China

**DOI:** 10.1038/srep02200

**Published:** 2013-07-16

**Authors:** Chuansong He, Shuwen Dong, M. Santosh, Xuanhua Chen

**Affiliations:** 1 Institute of Geophysics, China Earthquake Administration 100081, Beijing, China; 2 Chinese Academy of Geological Science, 100037, Beijing, China; 3 School of Earth Sciences and Resources, China University of Geosciences, 100083, Beijing, China

## Abstract

South China, composed of the Yangtze and Cathaysia Blocks and the intervening Jiangnan orogenic belt, has been central to the debate on the tectonic evolution of East Asia. Here we investigate the crustal structure and composition of South China from seismic data employing the *H-k* stacking technique. Our results show that the composition and seismic structure of the crust in the Jiangnan orogenic belt are identical to those of the Cathaysia Block. Our data reveal a distinct contrast in the crustal structure and composition between the two flanks of the Jiujiang-Shitai buried fault. We propose that the Jiujiang-Shitai buried fault defines a geosuture between the Yangtze and Cathaysia Blocks, and that the felsic lower crust of the Cathaysia Block and the Jiangnan orogenic belt may represent fragments derived from the Gondwana supercontinent.

The continental assembly of China, one of the cores of East Asia, is composed of the North China Craton, the Tarim Craton and the South China Block (Figure 1). The South China Block is composed of two sub-blocks, the Yangtze in the NW and Cathaysia in the SE (Figure 1), which collided and amalgamated during the Neoproterozoic, giving rise to the Jiangnan Orogen^1^,^2^,^3^,^4^,^5^,^6^,^7^,^8^,^9^,^10^. Collision zones such as this are critical to our understanding of orogenic process and the evolution of the continents^11^,^12^.

The South China continent abuts the western margin of the Pacific plate, and has witnessed plate subduction beneath the China mainland^13^. Since Grabau^14^, who first coined the term Cathaysia to describe the geology of southeastern China and part of the coastal region of West Pacific, debate has continued for over 80 years regarding the regional tectonics^13^, and the spatio-temporal evolution of the Cathaysia Block^14^,^15^,^16^. Several workers consider that the boundary (geosuture) between the Yangtze and Cathaysia Blocks is defined by the Shaoxing-Yichun-Pingxiang fault^6^,^17^,^18^.

In this study, we determine bulk crustal seismic properties and use these to infer the differences in structure and composition across the South China Block. Our study provides new seismic evidence for the precise location of the geosuture between the Yangtze Block and Cathaysia Blocks.

## Results

We used the *H*–*κ* stacking method to determine the average crustal thickness (*H*) and the ratio of *P*- and *S*-wave velocities (*Vp*/*Vs* ratio, or *κ*) under each station^19^. This method treats the crust as a single homogeneous layer, and constrains *H* and *κ* of the crust by searching the most energetic stack of the direct *P*s phase and multiples such as *PpPs*, *PsPs* + *PpSs* of the Moho according to the predicted delays relative to the incident *P* wave.

Before *H-k* stacking was performed, a reasonable bulk crustal *Vp* and search range of *H* and *k* were adopted. Based on deep seismic sounding investigation, the value of bulk crustal *Vp* in the study area should be approximately 6.3 *km s^−1^* 20. Therefore, we assume a mean crustal *P*-wave velocity (*V_P_*) of 6.3 *km s^−1^* and perform *H-k* stacking^19^,^21^ (search range: *H* = 25–50 *km*; *Vp/Vs* = 1.4–2.5). Finally, we obtained 251 estimates on robust crustal thickness and *Vp/Vs* ratios (Table S1). The resulting bulk crustal *Vp/Vs* ratios range from 1.53 to 2.11 with an average of 1.72. The Jiangnan orogenic belt and the Cathaysia Block are characterized by lower *Vp/Vs* ratio of 1.66–1.73, as compared to the *Vp/Vs* ratios of 1.74–1.79 in the SECCLMVZ (Southeast China coastal late Mesozoic volcanic zone). The *Vp/Vs* ratios of 1.76–2.11 in the Yangtze Block in Northwestern part of this study area are even higher (Figure 2, Table S1).

Our study shows that the average crustal thickness of South China is 33 *km.* However, locally the values vary widely between 25.5 and 56.9 *km*. Thinner crust of 25.6–32 *km* is observed in the Jiangnan orogenic belt and the Cathaysia Block, with a relatively flat Moho. A thick crust of 40–56.9 *km* is observed beneath the Yangtze Block in the northwestern part of the study area (Figure 3, Table S1).

## Discussion

For lower-crustal rocks, low *σ* (<0.26) (*Vp/Vs*<1.75), intermediate *σ* (0.26–0.28) and high *σ* (>0.28) (*Vp/Vs*>1.81) are characteristic of felsic, intermediate and mafic compositions^21^,^22^,^23^,^25^, respectively. If the bulk value of the entire crust is *σ*>0.28, the lower crust must have a value of ~0.30 and therefore, the low Poisson's ratio (*σ* = 0.25) for the Mesozoic-Cenozoic crust is considered to indicate a predominantly felsic compostion^21^. The *VP*/*VS* values are around 1.73 for felsic rocks, whereas for mafic rocks, this value tends to be greater than 1.73^22^,^23^,^24^. The values of bulk crustal *Vp/Vs* ratio are slightly less than that of lower crustal *Vp/Vp* ratio (about 0.02 or so)^21^,^22^,^24^,^25^. Therefore, we can use bulk crustal *Vp/Vs* ratio to estimate the lower crust *Vp/Vs* ratio^24^,^26^.

The distribution of bulk crustal *Vp/Vs* ratio indicates that the Jiangnan orogenic belt and the Cathaysia Block are characterized by felsic lower crust and the Yangtze Block by intermediate and mafic-ultramafic lower crust^21^,^25^. Notably, the bulk *Vp/Vs* ratio in the Jiangnan orogenic belt is lower than 1.70, which might be related to the collisional orogenesis. The similar crustal thickness on both sides of Shaoxing-Jiangshan-Pingxiang fault implies that the mountain root has been completely lost, or that the lower crust delamination beneath the Jiangnan orogenic belt was more intense, leading to the lower *Vp/Vs* values. The late Mesozoic coastal volcanic zone in Southeast China shows dominantly intermediate lower crust^21^,^25^, and the narrow distribution of these strips might reflect deep-seated magmatism associated with tectonic processes.

The distribution of the *Vp/Vs* presented here indicates that along the boundary of the Jiujiang-Shitai buried fault, there is a marked difference between the Jiangnan orogenic belt, the Cathaysia Block and Yangtze Block. The distribution of crustal thickness also assigns the Jiangnan-Shitai buried fault as the major boundary, with the crustal thickness increasing northwards in the Yangtze Block. The crustal thickness in the Jiangnan orogenic belt and the Cathaysia Block shows minor change laterally with a smooth variation of the Moho^28^ (Figure 3 and Figure 4). The depth domain profiles from our study at various stations bring out the presence of an important boundary in crustal structure between the BJT and AST (profile 1), HOJ and YOZ (profile 2), TAY and YIY (profile 3), and XNI and DUC stations (profile 4). Notably, this boundary line almost precisely coincides with the Jiangnan-Shitai buried fault. In figure 4, the crust beneath the Jiangnan orogenic belt and the Cathaysia Block along the red lines is characterized by a simple crustal structure. In contrast, the crust above the red lines, relating to the Yangtze Block, is dominantly featured by a complex crustal structure. A relatively large offset, of around 5 *km*, exists on both sides of the Jiujiang-Shitai buried fault above and below the red (or yellow) lines.

**Figure 1:**
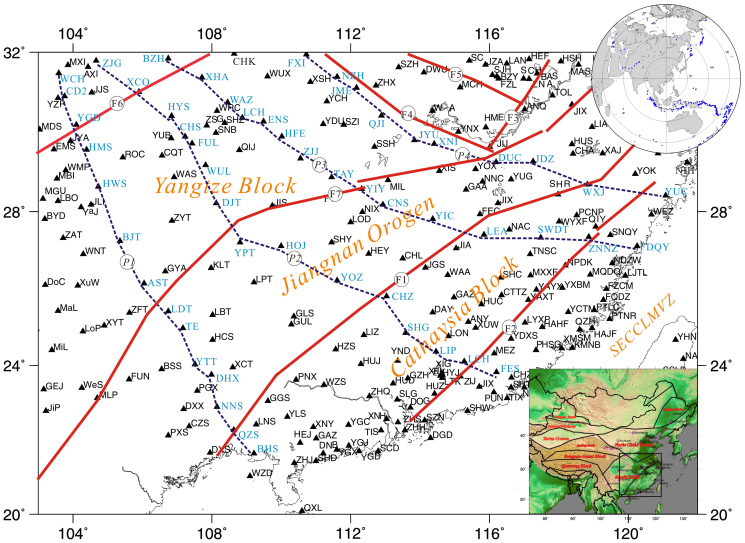
The distribution of seismic stations in South China and the general tectonic framework. F1: Shaoxing-Jiangshan-Pingxiang fault, F2: Zhenghe-Dapu fault, F3: Tanlu fault, F4: Xiangfan-Guangji fault, F5: Lu'an fault, F6: Longmenshan fault, F7: Jiujiang-Shitai buried fault^27^; *P1, P2, P3* and *P4*: profile, blue font: the seismic stations at the profiles; black triangle: seismic stations; SECCLMVZ: Southeast China coastal late Mesozoic volcanic zone. Inset figure (right-upper corner): Distribution of selected event. For each event-station pair, data were selected within the distance ranges of 30°–95° (The figure is generated using Generic Mapping Tool (http://gmt.soest.hawaii.edu/) by Chuansong He).

**Figure 2:**
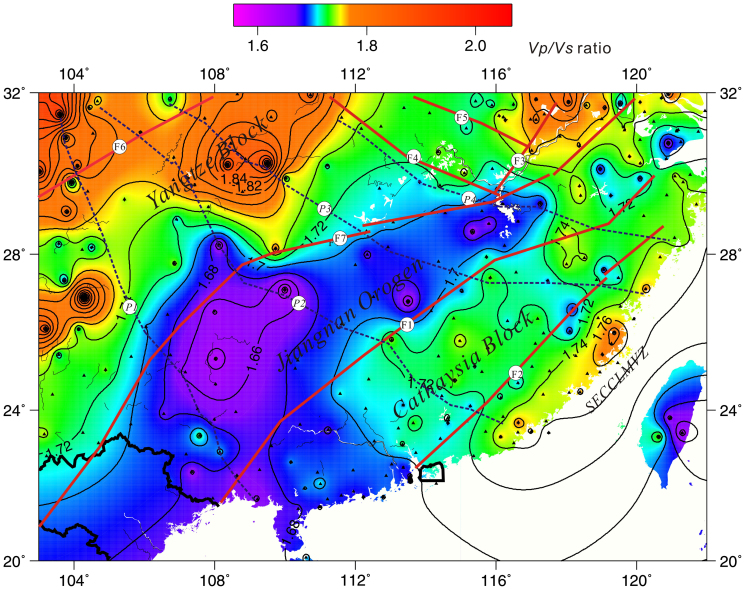
Distribution of *Vp/Vs* ratios in South China. F1: Shaoxing-Jiangshan-Pingxiang fault, F2: Zhenghe-Dapu fault, F3: Tanlu fault, F4: Xiangfan-Guangji fault, F5: Lu'an fault, F6: Longmenshan fault, F7: Jiujiang-Shitai buried fault^27^; *P1, P2, P3* and *P4*: profile; black triangle: effective data point; SECCLMVZ: Southeast China coastal late Mesozoic volcanic zone (The figure is generated using Generic Mapping Tool (http://gmt.soest.hawaii.edu/) by Chuansong He).

**Figure 3:**
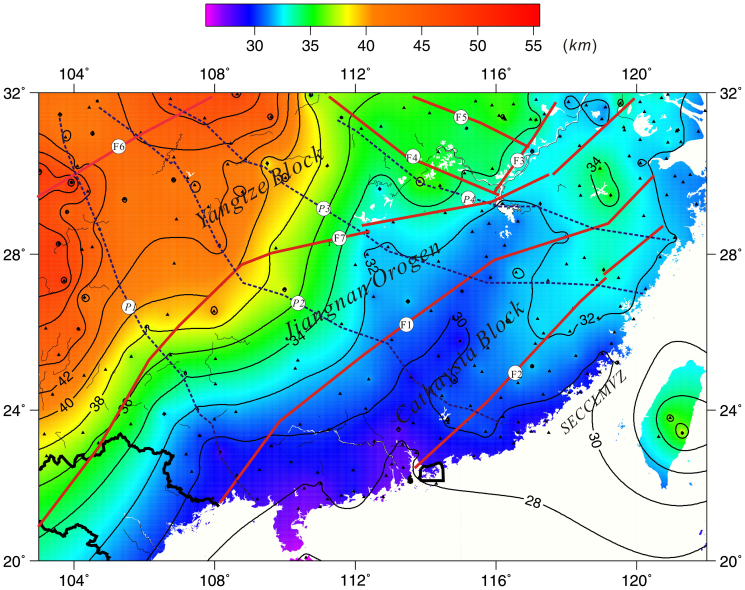
Distribution of crust thickness. F1: Shaoxing-Jiangshan-Pingxiang fault, F2: Zhenghe-Dapu fault, F3: Tanlu fault, F4: Xiangfan-Guangji fault, F5: Lu'an fault, F6: Longmenshan fault,F7: Jiujiang-Shitai buried fault^27^; *P1, P2, P3* and *P4*: profile; black triangle: effective data point, SECCLMVZ: Southeast China coastal late Mesozoic volcanic zone (The figure is generated using Generic Mapping Tool (http://gmt.soest.hawaii.edu/) by Chuansong He).

**Figure 4:**
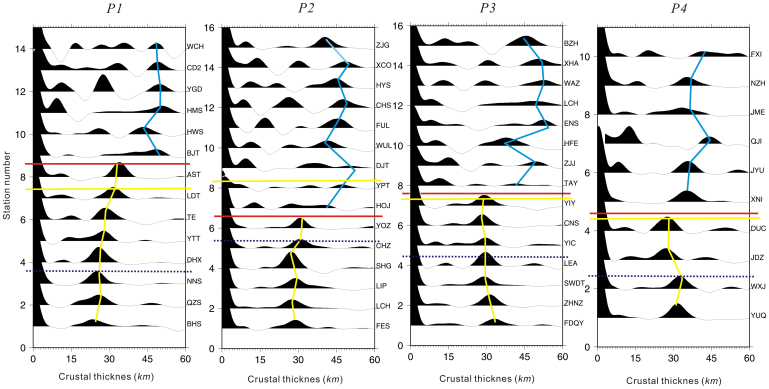
Profiles in the depth domain. *P1*: profile 1, *P2*: profile 2, *P3*: profile 3, *P4*: profile 4. Blue lines: the crust-mantle boundary of the Yangtze block, yellow lines: the crust-mantle boundary of the Jiangnan orogenic belt and the Cathaysia block, dotted line: the boundary between the Jiangnan orogenic belt and the Cathaysia block, yellow dotted line: Jiujiang-Shitai buried fault. The red line on all profiles almost precisely coincides with the Jiangnan-Shitai buried fault except for profile 2. The Jiangnan orogenic belt and the Cathaysia Block are characterized by a relatively flat Moho. The large offset between the Yangtze and Cathaysia Blocks lends support to the notion of collisional assembly of these two discrete blocks (The figure is generated using Generic Mapping Tool (http://gmt.soest.hawaii.edu/) by Chuansong He).

**Figure 5:**
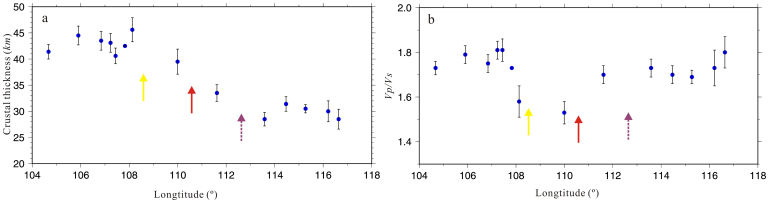
Blue dots and error bar represent the best estimates of crustal thickness (*h*) and *Vp/Vs* ratios (*k*) and errors of profile 2 in the *H–κ* stacking of receiver function. (a) Crustal thickness with error bars. (b) *Vp/Vs* ratios with error bars. Yellow arrow: Jiujiang-Shitai buried fault. Red arrow: the boundary between the Yangtz and Cathaysia Block from this studies, dotted arrow: the boundary between the Jiangnan orogenic belt and Cathaysia block (The figure is generated using Generic Mapping Tool (http://gmt.soest.hawaii.edu/) by Chuansong He).

Our results clearly indicate the absence of prominent differences in crustal structure on the two flanks of the Shaoxing-Jiangshan-Pingxiang fault (Figure 2, Figure 3, and Figure 4: above and below the dotted line). However, the Jiangshan-Shitai buried fault obviously defines an important boundary based on crustal composition and structure (Figure 1, Figure 2, Figure 3, Figure 4, Figure 5). Therefore, we propose that the Jiangshan-Shitai buried fault defines the boundary between the Yangtze and Cathaysia Blocks.

Recent studies have proposed that all the East and SE Asian continental terranes/blocks were directly or indirectly derived from the eastern margin of the Gondwana supercontinent^29^. There is increasing evidence to consider the South China block, including the Yangtze Block, as an integral part of East Gondwana in early Palaeozoic, rather than as a discrete continental block in the Palaeo-Pacific or a fragment of Laurentia^30^,^31^,^32^,^33^. If this model is true, the Jiangnan orogenic belt and the Cathaysia Block, with broadly consistent crustal characteristics and thickness, might represent fragments from the Gondwana assembly.

An active continental-margin model, with the subduction of the paleo-Pacific plate beneath the South China Craton, has been invoked in several studies to account for the extensive magmatic zone in southeastern China during the Mesozoic^3^,^34^,^35^,^36^,^37^,^38^. This has led to the popular concept that the Mesozoic low-angle subduction of the Pacific Plate has played a major role in the thinning of the South China Land Mass^39^,^40^.

Subduction and collision tectonics may generate local topography on Moho which may survive for a long time^41^,^42^. The dipping Moho topography is considered as an indication of the remnants of deep collision and subduction^43^. Our studies show little lateral variations in crustal thickness and a flat Moho in the Cathaysia block and Jiangnan orogenic belt. These results do not support the model of low-angle subduction of the Pacific Plate and resultant crustal thinning.

Delamination refers to the loss and sinking of the portion of the lower crust and (or) the lowermost lithosphere from the tectonic plate to which it was attached. This can occur when the lower crust and (or) the lower portion of the lithosphere becomes denser than the surrounding mantle. Because of the instability of higher density (e.g. thickening lower crust) material above lower density material, the lower crust and (or) the lower lithosphere separates from the tectonic plate and sinks into the mantle^44^,^45^,^46^, which results in asthenospheric upwelling into the space previously occupied by thickened lithosphere. Flow of hot mantle material encounters the base of the thin lithosphere and often results in melting and a new phase of volcanism^44^,^45^,^46^.

Since the Mesozoic, the South China region has been located at the center of a triangular area surrounded by westward subduction of the Pacific plate (Cenozoic, about ~50 Ma), northward subduction of the India Plate beneath the Eurasia Plate (Cenozoic, about ~50 Ma), and collision of the North and South China blocks along the Central China Orogen (Permian-Triassic, about 290–250 Ma)^28^. This region (including the Yangtze and Cathaysia blocks) thus marks the frontier of a super-convergent regime. Within the super-convergence domain, the compressional structures in the center of the South China Block are mainly characterized by shortening, thrusting and decollement^47^.

The collision between the Yangtze and Cathaysia Blocks generated a thick lithospheric root in the Hercynian-Indosinian period (409–205 Ma). Subsequently, in Yanshanian (208–135 Ma), delamination and asthenospheric upwelling led to extensive lithospheric extension and thinning^48^,^49^. The collision between the Yangtze and the Cathaysia Block in the Triassic as well as crustal detachment of the eastern Yangtze Block might have led to the thickening of lower crust and delimanition^50^.

The lower *Vp/Vs* ratio in the bulk lower crust of the Jiangnan orogenic belt and Cathaysia Block might suggest deep process such as lower crustal delamination, which resulted in the dominantly felsic lower crust^21^,^25^,^26^ beneath the Jiangnan orogenic belt and Cathaysia Block. Therefore, we favor lower crustal delamination of the Cathaysia Block and crustal thinning associated with mantle upwelling^44^,^45^,^46^,^51^ as the most plausible scenario to explain the extensive magmatic activity in this region. A recent *S* receiver function study indicates lithospheric thinning in Southern China^52^, which supports the delamination model for this area^53^,^54^. Therefore, we consider that the lower crust/lithosphere delamination^55^ beneath the Cathaysia Block might have led to lithospheric thinning in this area and the surrounding regions.

**Figure 6:**
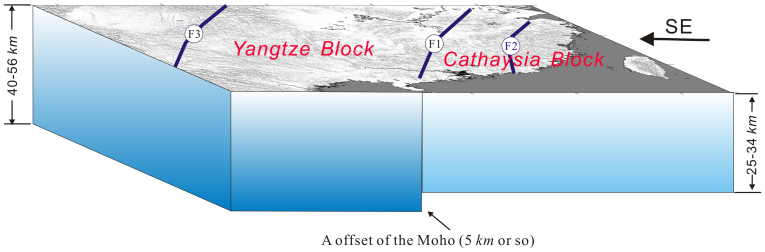
Collision model between the Yangtze and Cathaysia Blocks. F1: Jiujiang-Shitai buried fault, F2: Shaoxing-Jiangshan-Pingxiang fault, F3: Longmenshan fault. The geosuture between the Yangtze and the Cathaysia Blocks is defined by Jiujiang-Shitai buried fault. A relatively large offset, of around 5 km, exists on both sides of the Jiujiang-Shitai buried fault. The Cathaysia Block might suggest deep process such as lower crustal delamination, which resulted in the dominantly felsic lower crust and crustal thinning beneath the Jiangnan orogenic belt and Cathaysia Block. The Yangtze Block is characterized by intermediate and mafic-ultramafic lower crust (The figure is generated using Generic Mapping Tool (http://gmt.soest.hawaii.edu/) by Chuansong He).

In summary, we envisage the following processes: (1) tectonic thickening of the crust and lithosphere by convergence; (2) asthenospheric upwelling and infiltration of the lithosphere; (3) subsequent weakening of the lithosphere by this infiltration, resulting in delamination of the lower crust and mantle. The thickened lower crust was eclogitic, hence denser than the underlying mantle. The geosuture between the Yangtze and Cathaysia Blocks is defined by Jiujiang-Shitai buried fault. The Cathaysia Block, dominated by felsic bulk lower crust^21^,^24^,^25^, is probably a fragment of the Gondwana supercontinent. The large offset between the Yangtze and Cathaysia Blocks lends support to the notion of collisional assembly of these two discrete blocks (Figure 6).

## Methods

Teleseismic receiver functions^56^ are very sensitive to the S-wave velocity beneath the station and have proven to be a useful tool for estimating crustal thicknesses and *Vp/Vs* ratios beneath individual seismic stations^19^,^57^,^58^. The *P*-to-*S* converted phase at the Moho and the first reverberated phases in the crust are generally apparent in the receiver function waveforms, and their relative travel times can then be employed to constrain the crustal thickness and the bulk *Vp/Vs* ratio below the recording station^21^,^57^. Deciphering the geological evolution of the Earth's continental crust requires knowledge of its bulk composition and global variability^21^. Average *Vp/Vs* or Poisson's ratio (*σ* = 0.5[1−((*Vp/Vs*)^2^−1)^−1^]), can be used to complement petrological studies of crustal composition^58^.

This study performs a careful analysis of the receiver functions in South China, in order to characterize the bulk seismic properties of crust with local estimates for the crustal thickness and *Vp/Vs* ratio^19^,^21^. For this purpose, we apply the stacking procedure of Zhu and Kanamori^19^ (2000) to 281 seismic stations located in South China (Figure 1).

These stations have been in operation between from July 2008-present. We selected a total of 424 events with magnitude mb ≥ 6.0 recorded by those stations^59^ (Figure 1 and Table S2). For each event-station pair, data were selected within the distance ranges of 30°–95° and initially windowed 15 *s* before and 120 *s* after the *P*-wave pick. Only signals with a good signal-to-noise ratio and a clearly identifiable *P*-wave arrival were used. Data are filtered using a zero-phase Butterworth bandpass filter with corner frequencies of 0.03–3 *Hz*.

In our study we use a modified frequency domain deconvolution^56^,^60^, which is implemented by dividing the spectrum *R(ω)* of the teleseismic *P* waveform by the source spectrum *S(ω)*: 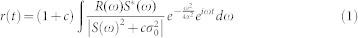
Where S*(ω) is the complex conjugate of *S(ω)*, the Gaussian-type low-pass filter 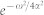
is added to remove high-frequency noise. The quantity 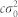 is used to suppress “holes” in the spectrum *S(ω)*, thus stabilizing the deconvolution where *r(t)* is the radial receiver function. The 1 + *c* factor is used to compensate the amplitude loss due to the water level^19^. In this study, the Gaussian factor and water level were set to 3 and 0.01, respectively.

The time separation between *Ps* and *P* can be used to estimate crustal thickness, given the average crustal velocities, 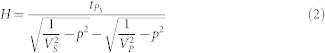
Where *p* is the ray parameter of the incident wave and the crustal velocity is given, we can obtain the thickness estimation; however, one problem is the trade-off between the thickness and crustal velocities. 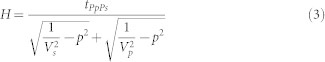

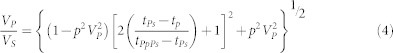


The trade-off influence can be reduced by using the later phases, which provide additional constraints. The precise crustal *H* and *Vp/Vs* ratios can be estimated by (3) and (4)^19^,^21^.

The stacking is usually done in the time domain for a cluster of events^60^. We propose a straightforward *H-k* domain stacking defined as below: 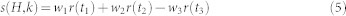
Where *r(t)* is the radial receiver function, *t_1_*,*t_2_*and *t_3_* are the predicted *Ps*, *PpPs* and *PpSs + PsPs* arrival time corresponding to crustal thickness *H* and *Vp/Vs* ratios (ratio *k*)*,* as given in (2)–(4). The *w_i_* are weighting factors, and ∑*w_i_* = 1. The *s(H,k)* reaches a maximum when all of these phases are stacked coherently with the correct *H* and *k*^21^. Here, we chose unequal weights (0.6, 0.3 and 0.1) for *Ps*, *PpPs* and *PpSs* + *PsPs* phases of the Moho, respectively. Using the Taylor expansion of *s(H,k)* at the maximum and omitting the higher-order terms, one gets the variances of *H* and *k*: 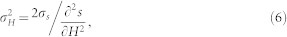

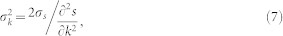


Where 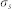 is the estimated variance of 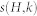 from stacking^19^. On the other hand, the study shows that the uncertainty of *H* is <0.5 *km* for a 0.1 *km/s* uncertainty in *Vp*^19^.

To quantitatively estimate the uncertainty of our results, we measured error bars for *h* and *κ* of the profile 2, which line up with longitude, by taking into account uncertainties (Figure 5). Their uncertainties are estimated using (6) and (7)^19^. Here we show two examples of *H-k* stacking computed at two stations located at the study area (Figure S1).

We migrate the receiver functions to depth using the iasp91 velocity model^61^. The amplitude itself is proportional to the velocity or, more precisely, the impedance contrast at that location. Based on it, we converted the time domain receiver functions into the depth domain after corrections for the incidence angle effect (correcting the move out of *Ps* conversions to same incidence or near vertical incidence)^62^.

By stacking all depth domain receiver functions from different backazimuths at each station respectively, we obtain the average receiver function at each station (multi-events to stack their receiver function)^60^. The average (stacked) receiver function can clearly delineate Moho interface which is usually the largest discontinuity^63^. We then get a rough estimation of the Moho depth variation beneath each station from the average receiver function (Figure S2–S3).

## Author Contributions

C.S.H. wrote the main manuscript text and S.W.D., M.S. and X.H.C. revised the manuscript. All authors reviewed the manuscript.

## Supplementary Material

Supplementary Information Supplementary information
