# Supplementary material for: Seismic Evidence for a Geosuture between the Yangtze and Cathaysia Blocks, South China
Source: Sci Rep. 2013 Jul 16;3:2200. doi: 10.1038/srep02200 (PMC3712313; doi:10.1038/srep02200)
Supplement: Supplementary Information [file srep02200-s1.pdf]

Seismic Evidence for a Geosuture between the Yangtze and Cathaysia Blocks, South China

By

Chuansong He[1], Shuwen Dong[2], M. Santosh[3], Xuanhua Chen[2]

From

[1] Institute of Geophysics, China Earthquake Adminstration 100081, Beijing, China

[2]Chinese Academy of Geological Science, 100037, Beijing, China

[3]School of Earth Sciences and Resources, China University of Geosciences Beijing, 29 Xueyuan Road, Beijing 100083, China

***Correspondence**: Chuansong He
Institute of Geophysics, China Earthquake Adiminstration, 100081, Beijing, China
Tel.: +86 10 68729303; Fax: +86 10 83552767.
E-mail address: hechuansong@yahoo.com.cn (C.-S. He).

Table S1 **Results of *h-k* stacking.** Station: seismic station; *n*: the number of receiver function used by the *h-k* stacking; t_{ps}: the arrival time of the Moho converted phase; *h*: crustal thickness (*km*); ⊿*h*: uncertainty range of stacking result (*h*); *k*: *Vp/Vs* ratio; ⊿*k*: uncertainty range of stacking result (*k*); *Vp*: the average *P* velocity of the crust used by the *h-k* stacking; *q* describes the quality of the resultant stack, we ranked the stations into three categories: 'best' is 1, 'good' is 2 and 'fair' is 3. When *Ps* and *PpPms* can be clearly seen, *PpSms* can be divided into 3 categories: "can be clearly seen", "can be seen" and can't be seen, based on it, the stations are divided three categories: "best", "good" and "fair".

Station	*n*	t_{ps}	*h*	⊿*h*	*k*	⊿*k*	*Vp*	*q*
ANQ	89	4	30.5	1.9	1.79	0.05	6.3	1
BAS	51	4	32.4	1.8	1.75	0.06	6.3	1
BZY	28	4.5	37	1.8	1.73	0.04	6.3	3
FZL	99	4.6	35.9	1.3	1.78	0.04	6.3	1
HEF	86	4.3	31.5	1.3	1.82	0.04	6.3	2
HSH	21	4.5	30.9	1.6	1.89	0.07	6.3	2
HUS	81	4.3	33.5	4.8	1.77	0.19	6.3	1
JIX	89	4.2	33	1	1.77	0.04	6.3	1
JZA	87	4.1	35.6	3.2	1.69	0.08	6.3	1
LAN	93	4.1	35.9	1.5	1.7	0.04	6.3	1
LNA	30	5	37.5	1.2	1.81	0.04	6.3	2
MAS	85	3.9	31.9	0.8	1.73	0.03	6.3	1
SCH	57	4.3	33.6	1.3	1.77	0.04	6.3	1
SJH	30	4.6	35.9	1.4	1.78	0.05	6.3	2
TOL	87	3.9	31.6	2.4	1.75	0.07	6.3	1
CHK	150	6.5	50	0.1	1.78	0	6.3	3
CHS	168	5.8	43.1	1.8	1.81	0.04	6.3	2
FUL	176	5.4	40.6	1.5	1.81	0.05	6.3	3
QIJ	179	5.8	43.5	2	1.81	0.06	6.3	2
ROC	200	4.9	40.4	1.5	1.73	0.04	6.3	1
SHZ	158	6.7	40.1	2.5	2.01	0.11	6.3	2
WAS	201	4.9	43.6	1.9	1.68	0.04	6.3	1
WAZ	65	6.4	44.4	1.5	1.87	0.04	6.3	3
WUL	164	5.1	42.5	0.1	1.73	0	6.3	3
WUX	149	6.3	49.1	1.7	1.77	0.03	6.3	3
YUB	212	5.3	41	1.2	1.78	0.03	6.3	1
DSXP	74	3.5	28	1.7	1.75	0.07	6.3	1
FDQY	74	4.1	30.9	1.5	1.8	0.06	6.3	1
FQDZ	71	3.9	29.6	1.2	1.8	0.04	6.3	1
FZCM	63	4.3	31.1	1.7	1.84	0.06	6.3	1
HAHF	75	3.8	31.5	1.4	1.74	0.05	6.3	1
HAJF	77	3.5	28	1.1	1.76	0.05	6.3	1
LJTL	76	3.9	32	1	1.73	0.04	6.3	1
LYXP	26	3.9	32.6	1.3	1.72	0.04	6.3	2
MQDQ	77	4.1	32.4	1.2	1.76	0.04	6.3	1
MXXF	77	4	32.5	1.2	1.75	0.05	6.3	2

NDZW	82	4	32.5	1.3	1.74	0.05	6.3	1
NPDK	75	3.5	32.5	1.5	1.64	0.04	6.3	2
PCNP	74	4.1	33	1.5	1.76	0.05	6.3	1
PHSG	74	3.9	30.5	1.6	1.76	0.05	6.3	1
PTLC	61	3.8	29	1.5	1.8	0.07	6.3	1
PTNR	66	3.6	29.5	1.2	1.74	0.04	6.3	1
QZH	34	3.7	29	0.8	1.76	0.04	6.3	1
SNQY	77	3.8	33.1	2	1.69	0.08	6.3	2
SWDT	84	3.7	32.1	1.1	1.69	0.04	6.3	1
WYXF	79	4.1	32.5	2.1	1.77	0.08	6.3	3
XMSM	26	3.7	29.5	1.4	1.75	0.06	6.3	1
YAXT	81	3.9	31.9	0.9	1.73	0.04	6.3	2
YAYX	81	3.9	32.5	1	1.72	0.04	6.3	1
YCTM	47	4	32	1.3	1.76	0.05	6.3	2
YDXS	76	3.6	32	1.1	1.69	0.04	6.3	1
YXBM	81	3.6	32.6	1.4	1.66	0.05	6.3	1
ZHNZ	78	3.9	32.6	1.9	1.73	0.06	6.3	1
CHZ	64	3.8	28.5	1.9	1.8	0.07	6.3	1
DGD	41	3.3	28	1.7	1.72	0.06	6.3	1
DNB	75	3.2	27.5	1	1.7	0.04	6.3	1
DOG	50	3.2	27.1	1.2	1.71	0.05	6.3	1
FES	36	3.6	30	2	1.73	0.08	6.3	1
GAZ	22	3.3	27	1.5	1.73	0.06	6.3	1
GZH	31	3.7	30	1.4	1.75	0.05	6.3	1
HEJ	33	3.2	27	1.2	1.71	0.04	6.3	1
HUD	30	3.2	27	1.2	1.71	0.05	6.3	1
HUJ	62	3.6	30.5	1.4	1.71	0.05	6.3	1
HUZ	32	3.4	29.4	1.3	1.7	0.04	6.3	1
HYJ	65	3.7	29.9	2.2	1.74	0.07	6.3	1
JIX	14	3.8	31.1	1.5	1.73	0.05	6.3	2
LCH	42	3.5	30.5	0.8	1.69	0.03	6.3	1
LIP	47	3.6	31.4	1.4	1.7	0.04	6.3	1
LIZ	35	3.8	32	1.6	1.72	0.04	6.3	1
LTK	54	3.4	30.5	1.9	1.67	0.07	6.3	1
MEZ	61	3.5	30	0.8	1.71	0.03	6.3	1
NAO	38	3.6	28	1.4	1.78	0.05	6.3	1
NAP	7	3.3	28	1.9	1.71	0.07	6.3	2
PUN	49	3.9	30	1	1.78	0.04	6.3	1
SCD	53	3.1	26.5	1.8	1.71	0.07	6.3	1
SHD	53	3.1	27.5	1.4	1.67	0.05	6.3	1
SHG	40	3.4	28.5	1.3	1.73	0.04	6.3	1
SHT	58	3.6	29.5	1.2	1.74	0.04	6.3	2
SHW	32	3.4	28.5	1.4	1.72	0.06	6.3	2
SLG	31	3.4	27.4	0.9	1.74	0.04	6.3	1

SZN	34	3.6	29.5	1.2	1.73	0.05	6.3	1
TIS	55	3.3	27.9	1.5	1.7	0.06	6.3	1
TIX	38	3.6	29.5	1.1	1.74	0.05	6.3	1
XFJ	60	3.7	30.9	1.2	1.72	0.04	6.3	2
XIG	43	3.6	30	1.1	1.73	0.04	6.3	2
XNH	68	3.2	27.5	1.5	1.69	0.05	6.3	1
XNY	56	3.4	30.1	1.1	1.69	0.04	6.3	1
YGC	52	3.5	30.1	1.4	1.7	0.05	6.3	1
YGD	31	3.1	28	2	1.67	0.07	6.3	2
YGJ	70	3.4	30.5	1.8	1.68	0.05	6.3	1
YGX	49	3.4	29.5	1	1.68	0.04	6.3	1
YND	43	3.4	29.6	1.3	1.69	0.05	6.3	1
ZHH	19	3.1	28	2	1.66	0.07	6.3	1
ZHJ	41	3	27	1.8	1.67	0.06	6.3	2
ZHQ	101	3.2	27.5	1.3	1.7	0.05	6.3	1
ZHS	53	3.1	27.5	1.5	1.67	0.05	6.3	1
ZIJ	71	3.6	31	1.3	1.71	0.04	6.3	1
BHS	127	3	28.5	1.3	1.64	0.05	6.3	1
BSS	108	3.4	31	1.5	1.66	0.04	6.3	1
CZS	143	3.2	30.9	1.2	1.63	0.04	6.3	1
DHX	122	3.3	30.5	1.1	1.65	0.04	6.3	1
DXS	116	3.2	28.6	1	1.68	0.04	6.3	1
DXX	146	3.4	30.1	1.7	1.69	0.05	6.3	1
GGS	118	3.2	28.5	1.2	1.68	0.05	6.3	1
GLS	43	3.7	32	1.4	1.7	0.05	6.3	1
GUL	90	3.7	32.4	1.2	1.69	0.04	6.3	1
HCS	158	3.5	33	1.3	1.63	0.04	6.3	1
HZS	130	3.7	31	1.1	1.72	0.04	6.3	1
LNS	149	3.3	29.5	1.1	1.67	0.04	6.3	1
NNS	144	3.2	26.6	1.4	1.73	0.06	6.3	1
PGX	142	3.8	29.5	1.4	1.77	0.06	6.3	1
PNX	110	3.4	30	0.9	1.68	0.04	6.3	1
PXS	66	3.6	30.5	1.6	1.7	0.04	6.3	1
QZS	98	3.2	30	1.7	1.65	0.06	6.3	2
TE	79	3.6	33.5	0.9	1.64	0.03	6.3	1
WZS	133	3.3	30	1.2	1.66	0.05	6.3	1
XCT	140	3.5	32	1.3	1.66	0.04	6.3	1
YLS	139	3.5	30	1.5	1.71	0.04	6.3	1
YTT	148	3.5	31.6	1.6	1.67	0.05	6.3	1
AST	23	4.2	37	2.1	1.69	0.05	6.3	3
DJT	58	4.4	45.6	2.3	1.58	0.07	6.3	2
KLT	18	4.3	42.5	1.5	1.61	0.06	6.3	3
LBT	54	3.4	34.9	1.6	1.59	0.06	6.3	1
LDT	70	4	35.5	2.2	1.68	0.05	6.3	1

WNT	121	6.8	37.5	2.3	2.1	0.09	6.3	2
XYT	35	4.7	37.9	1.3	1.75	0.05	6.3	3
ZFT	56	4.2	35.4	1.1	1.71	0.04	6.3	2
ZYT	80	5.1	40.1	2.2	1.77	0.05	6.3	3
SC	243	4.1	34.9	2	1.71	0.06	6.3	1
DWU	66	4.2	34.9	2	1.73	0.05	6.3	2
ENS	12	6.3	35.5	2	2.08	0.08	6.3	3
FXI	81	5	34.1	1.4	1.88	0.05	6.3	2
HFE	52	4.6	41.4	2.7	1.67	0.07	6.3	2
HME	69	4.1	35.5	1.8	1.7	0.05	6.3	1
JME	68	4.2	34.9	1.4	1.73	0.06	6.3	1
JYU	130	4.5	38.9	2.8	1.69	0.06	6.3	1
LCH	75	6	42	2.2	1.86	0.09	6.3	2
MCH	104	3.9	33.4	1.3	1.7	0.04	6.3	1
NZH	113	4.4	35.4	2.6	1.75	0.08	6.3	2
SSH	115	3.5	30.4	1.4	1.7	0.05	6.3	1
SZH	132	4.3	35.9	1.6	1.72	0.05	6.3	2
WHA	30	4.3	34.5	2.6	1.76	0.1	6.3	3
WHN	87	4	35.9	1.6	1.67	0.06	6.3	2
XNI	106	4.5	37.1	1.2	1.73	0.03	6.3	1
YDU	128	4.4	38.5	2.4	1.69	0.09	6.3	2
YNX	84	4.3	33.5	2.7	1.78	0.07	6.3	2
ZHX	121	4.1	33.6	1.9	1.74	0.06	6.3	2
QXL	41	3	25.5	1.1	1.72	0.05	6.3	1
CHL	40	3.2	32.6	1.8	1.59	0.07	6.3	1
CHZ	45	3.9	30	0.1	1.78	0	6.3	2
CNS	35	3.6	31.5	2	1.68	0.06	6.3	1
HEY	46	3.5	30.4	1.2	1.69	0.04	6.3	1
HOJ	23	3.5	39.5	2.4	1.53	0.05	6.3	3
JIS	31	5	36	1.6	1.85	0.06	6.3	2
LOD	34	3.8	32.5	1.4	1.7	0.04	6.3	1
MIL	33	3.7	32	1.2	1.71	0.04	6.3	1
NIX	44	3.4	31	1	1.65	0.04	6.3	1
YIY	50	3.7	31	1.5	1.72	0.05	6.3	1
YOZ	46	3.9	33.5	1.6	1.7	0.04	6.3	1
CS	92	4.1	33.5	1.9	1.73	0.05	6.3	1
CZ	17	4.1	32	1.9	1.77	0.06	6.3	3
GC	73	3.8	30.1	1.3	1.76	0.06	6.3	1
JT	66	3.8	35.9	2.5	1.64	0.06	6.3	1
LIS	46	4.1	29.5	1.6	1.83	0.07	6.3	1
NT	73	4.5	32	1.6	1.85	0.06	6.3	1
WX	90	4.1	32	1.2	1.77	0.04	6.3	1
YX	27	4.1	33.5	1.1	1.73	0.04	6.3	2
ANY	83	3.7	31.5	1.7	1.71	0.05	6.3	1

DAY	61	3.5	29.4	1.3	1.72	0.04	6.3	1
DUC	59	3.4	31	1.6	1.67	0.05	6.3	1
FEC	110	3.7	30.5	1.2	1.73	0.04	6.3	1
GAA	43	3.2	30.9	2	1.63	0.09	6.3	2
GAZ	111	3.6	27.6	1.4	1.78	0.05	6.3	2
HUC	89	3.9	31	1.4	1.75	0.06	6.3	1
JDZ	88	3.4	31.9	1.5	1.64	0.06	6.3	1
JGS	125	3.6	31.1	1.7	1.7	0.07	6.3	1
JIA	117	3.4	29.5	1.3	1.69	0.04	6.3	1
JIJ	133	3.9	34	0.1	1.7	0	6.3	1
JIX	116	3.4	30.4	1.3	1.68	0.04	6.3	1
LEA	18	3.7	29.6	1.8	1.76	0.06	6.3	2
LON	62	3.7	29.4	2	1.76	0.08	6.3	1
NAC	103	3.9	33.1	2.2	1.72	0.06	6.3	1
NNC	62	3.3	30.5	1.3	1.65	0.05	6.3	1
SHC	46	3.8	32.5	1.3	1.7	0.05	6.3	1
SHR	115	4	31	1.9	1.79	0.06	6.3	1
WAA	121	3.6	29.5	1.7	1.74	0.06	6.3	1
XIS	143	3.7	33	1.2	1.68	0.04	6.3	1
XUW	103	3.8	31.5	1.7	1.73	0.06	6.3	1
YIC	98	3.7	32	1.2	1.7	0.04	6.3	2
YOX	115	3.7	32.4	1.5	1.7	0.04	6.3	2
YUG	130	3.3	30.1	1.4	1.67	0.06	6.3	1
AXI	131	5.2	43	1.9	1.72	0.04	6.3	1
BZH	141	5.5	48.4	2.6	1.68	0.05	6.3	2
CD2	73	4.2	38.6	1.8	1.66	0.04	6.3	3
HMS	146	6.1	47	2.2	1.78	0.04	6.3	2
HWS	137	5.2	41.5	1.4	1.76	0.04	6.3	1
HYS	134	5.4	43.5	1.8	1.75	0.04	6.3	1
JJS	112	5.5	41	1.7	1.82	0.04	6.3	2
JLI	134	5	46	2.4	1.65	0.05	6.3	2
JYA	102	5.7	56.9	2	1.6	0.04	6.3	2
LBO	131	5.9	52.5	4	1.68	0.07	6.3	2
MDS	82	8.3	52.1	2	1.96	0.05	6.3	3
WCH	142	7.5	41.1	1.9	2.11	0.06	6.3	3
WMP	151	5.9	40.4	2	1.89	0.05	6.3	1
XCO	149	5.8	44.5	1.8	1.79	0.04	6.3	1
XHA	104	6.1	47.9	2	1.77	0.04	6.3	1
YGD	129	6	41	2.9	1.89	0.07	6.3	2
ZJG	141	5	41.4	1.4	1.73	0.03	6.3	1
DOT	20	3.8	29.5	2.6	1.77	0.08	6.3	1
HUH	50	4.1	32	2.8	1.78	0.07	6.3	1
NAH	21	3.6	30.9	1.4	1.7	0.05	6.3	3
QHS	51	3.7	32.5	1.1	1.68	0.03	6.3	1

SSE	45	3.9	33	1.5	1.71	0.05	6.3	1
TMS	53	3.9	32.4	1.4	1.72	0.05	6.3	1
TPS	78	3.9	32.5	1.4	1.72	0.04	6.3	1
XKS	42	3.9	32	1.4	1.73	0.05	6.3	1
KMNB	16	3.8	29	1.2	1.78	0.05	6.3	1
SSLB	15	4.1	39.1	2.7	1.62	0.07	6.3	3
TPUB	9	4.3	33.5	1.8	1.78	0.05	6.3	3
YULB	15	4	40.4	3.8	1.59	0.08	6.3	2
DoC	84	7.2	45.6	2.1	1.96	0.06	6.3	3
FUN	86	4.1	35.5	1.2	1.7	0.04	6.3	1
GEJ	95	4.9	39	1.4	1.76	0.04	6.3	1
GYA	74	4.5	40.1	2	1.68	0.05	6.3	1
JiP	119	4.4	37.5	1.2	1.71	0.04	6.3	1
LoP	119	4.9	40.5	1.5	1.72	0.04	6.3	1
MaL	113	5.2	46.9	4.6	1.67	0.1	6.3	1
MiL	95	5.2	42.4	1.5	1.74	0.04	6.3	1
MLP	72	4.2	34.9	1.5	1.72	0.05	6.3	1
WeS	110	4.5	37.9	1.2	1.72	0.03	6.3	1
XuW	105	5.3	48	1.7	1.67	0.03	6.3	1
YaJ	103	5.6	45.6	3.1	1.74	0.06	6.3	1
ZAT	122	5.6	55.5	2.2	1.6	0.06	6.3	2
CHA	59	4.2	33	1.4	1.77	0.05	6.3	1
CHX	64	4.1	32.1	2.7	1.78	0.08	6.3	1
HAY	77	3.5	30.9	2.9	1.69	0.08	6.3	1
HAZ	70	4.1	31.4	1.8	1.78	0.06	6.3	1
HUZ	77	3.9	32	1	1.73	0.03	6.3	1
JAX	58	3.2	33.5	1.2	1.58	0.04	6.3	1
LIA	70	3.9	37	1.8	1.63	0.06	6.3	1
NIB	66	3.8	31.4	1.2	1.73	0.04	6.3	1
NIH	39	3.9	31	2	1.76	0.07	6.3	1
QIY	38	3.8	35	1	1.66	0.03	6.3	1
WEZ	30	4	31.9	1.4	1.76	0.05	6.3	1
XAJ	18	4.2	37	1.9	1.68	0.06	6.3	3
XIC	50	3.8	31.5	2	1.73	0.07	6.3	1
XSH	70	3.6	32.5	3	1.66	0.07	6.3	1
YIX	54	3.9	31.1	1.5	1.75	0.05	6.3	1
YOK	44	4	32.4	1.8	1.74	0.05	6.3	1
YUQ	56	3.9	32.5	1.4	1.73	0.05	6.3	1
YUY	63	4	31	0.9	1.77	0.04	6.3	1

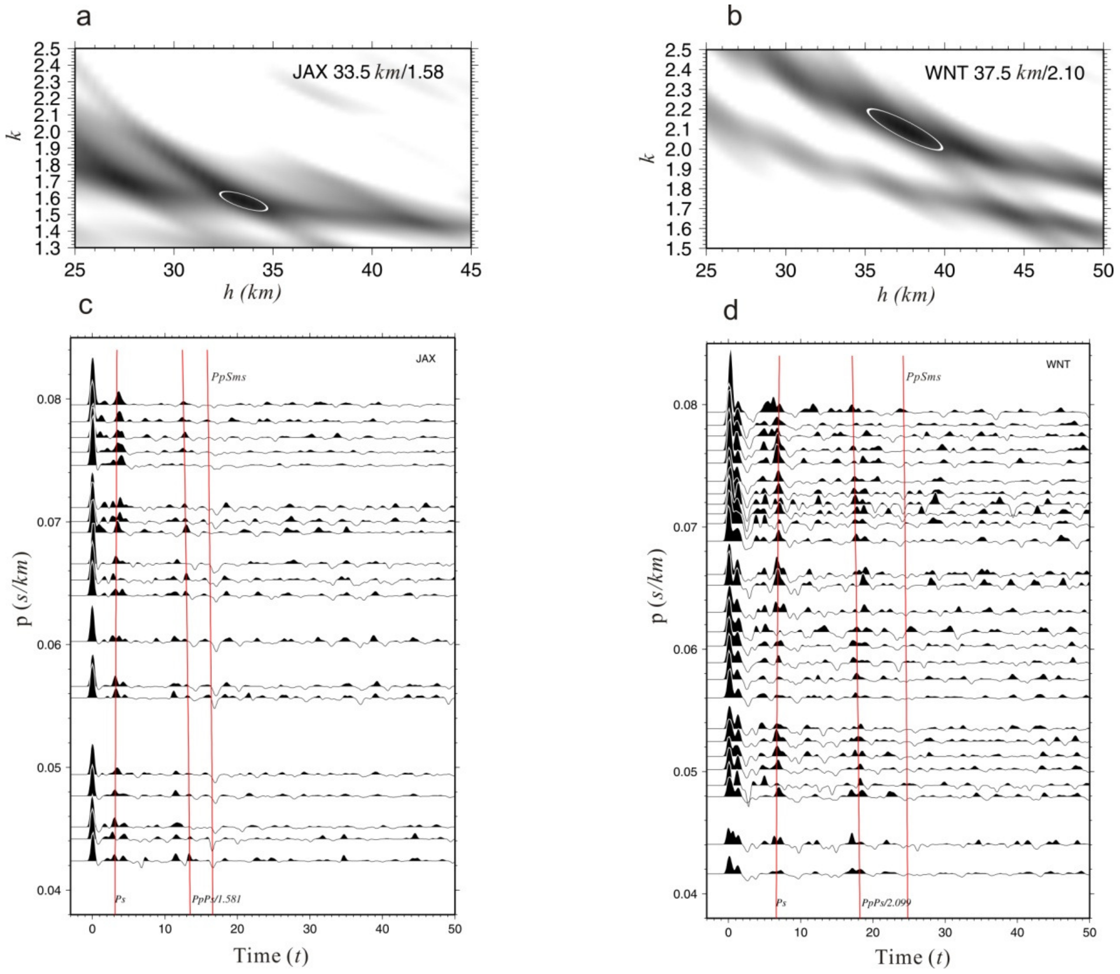

Figure S1 | ***H-κ* stacks from stations JAX and WNT.** *p*: slowness, *h*: Moho depth, *k: Vp/Vs* ratio; The maximum value of each stack is marked by the open oval. For first group (a), the estimated Moho depth is 33.5 *km*, with an uncertainty range of [32.3, 34.7]. *Vp/Vs* ratio, *k*, is 1.58, uncertainty range [1.54, 1.62] ; for the second group (b), the estimated Moho depth is 37.5 *km*, with uncertainty range [35.2, 39.8], *Vp/Vs* ratio, *k*, is 2.1, uncertainty range [2.01, 2.19]. The *P*-to-*S* converted phase (*Ps*) at the Moho and the first multiply reverberated phases (*PpPms* and *PpSms*) in the crust are clearly apparent in the receiver function waveforms (c, d), and their relative traveltimes can then be employed, more precisely, to constrain the crustal thickness and the *Vp/Vs* ratio below the recording station (c, d).

Table S2 **Earthquake catalogues used by this study.** *YMD*: year, month, day; *Jday*: Julday; *h*: hour; *m*: minute; *s*: second; *long*: longitude; *lat*: latitude; *d*: focal depth.

YMD	*Jday*	*h*	*m*	*s*	*long*	*lat*	*d*
20070802	214	3	21	42.82	−179.97	51.31	21
20070805	217	9	28	39.83	168.72	−19.15	40
20070808	220	17	4	57.84	107.68	−5.93	291
20070808	220	17	5	4.92	107.42	−5.86	280
20070812	224	12	5	19.85	166.27	−11.38	42
20070815	227	20	22	11.12	−177.55	50.32	9
20070816	228	4	3	3.5	−177.57	50.24	14
20070816	228	8	39	28.44	159.46	−9.83	15
20070817	229	2	16	30.64	129.47	−5.3	21
20070817	229	3	4	3.09	129.43	−5.26	10
20070826	238	12	37	31.19	−174.34	−17.46	127
20070902	245	1	5	18.15	165.76	−11.61	35
20070902	245	2	35	23.65	165.7	−11.77	36
20070912	255	11	10	26.83	101.37	−4.44	34
20070912	255	14	40	5.73	101.46	−3.16	35
20070912	255	16	37	3.92	101.4	−3.14	35
20070912	255	23	49	3.72	100.84	−2.62	35
20070913	256	2	30	3.3	99.67	−1.69	28
20070913	256	3	35	28.72	99.63	−2.13	22
20070913	256	16	9	16.87	101.52	−3.17	53
20070914	257	6	1	32.27	101.17	−4.07	23
20070914	257	6	3	16.53	100.95	−4.42	25
20070914	257	11	51	30.19	179.68	−23.65	552
20070919	262	7	27	50.7	100.89	−2.75	35
20070920	263	8	31	14.49	100.14	−2	30
20070925	268	5	16	1.12	180	−30.97	416
20070926	269	12	36	26.89	153.5	−4.99	40
20070926	269	15	43	1.44	99.49	−1.79	26
20070927	270	19	57	44	169.28	−21.1	9
20070928	271	1	1	49.18	169.36	−21.21	12
20070928	271	1	35	51.97	169.37	−21.13	10
20070928	271	11	12	40.51	169.19	−21.4	10
20070928	271	11	16	39.15	169.14	−21.33	10
20070929	272	5	37	7.26	95.52	2.9	35
20070930	273	2	8	30.17	145.72	10.45	14
20070930	273	5	23	34.07	164.12	−49.27	10
20070930	273	9	47	51.97	164.11	−49.14	18
20071001	274	13	50	24.74	148.22	−3.16	10
20071002	275	3	43	38.91	101.21	−4.24	22
20071002	275	18	0	6.87	−161.71	54.51	32
20071004	277	12	40	31.13	92.9	2.54	35

20071005	278	7	17	52.81	179.46	-25.19	509
20071009	282	15	3	41.21	152.89	-4.81	39
20071010	283	0	19	16.79	99.48	-1.74	27
20071013	286	17	45	53.18	169.2	-21.23	37
20071015	288	12	29	34.86	167.55	-44.8	18
20071015	288	21	28	23.68	167.46	-44.79	19
20071016	289	21	5	43.27	179.53	-25.77	509
20071021	294	10	24	52.06	154.77	-6.31	46
20071023	296	19	56	47.39	99.9	-2	30
20071024	297	21	2	50.61	101.02	-3.9	21
20071025	298	13	50	4.26	154.23	46.01	20
20071031	304	13	44	19.76	-178.38	51.42	28
20071102	306	13	30	44.02	-173.07	-15.59	10
20071110	314	1	13	29.35	161.32	-51.78	10
20071110	314	23	19	43.32	100.53	-3.28	15
20071115	319	17	18	21.24	-175	-23	37
20071119	323	0	52	12.51	-178.75	-21.18	558
20071119	323	15	20	3.7	173.49	-21.97	10
20071119	323	20	32	48.8	-127.41	43.54	10
20071120	324	12	52	59.03	155.67	-6.91	52
20071120	324	15	28	24.89	-177.94	-29.99	32
20071122	326	8	48	27.53	147.1	-5.76	53
20071122	326	23	2	12.96	95.06	4.74	49
20071123	327	1	26	47.44	151.87	-4.63	150
20071125	329	2	51	57.22	101.16	-2.81	55
20071125	329	16	2	15.75	118.37	-8.29	20
20071125	329	17	41	38.04	100.41	-2.24	35
20071125	329	19	53	5.47	118.47	-8.22	18
20071127	331	11	49	58.01	162.15	-10.95	16
20071201	335	1	44	31.95	97.88	1.98	44
20071203	337	21	52	45.58	148.46	-6.37	29
20071209	343	7	28	20.82	-177.51	-26	152
20071212	346	23	39	59.79	-131.55	52.1	10
20071213	347	15	51	27.21	-172.37	-15.21	17
20071215	349	8	3	15.79	127.47	-7.53	175
20071215	349	9	39	53.62	131.09	-6.62	57
20071219	353	9	30	27.93	-179.51	51.36	34
20071219	353	11	34	56.95	155.78	-6.95	45
20071220	354	7	55	15.84	178.29	-39.01	20
20071221	355	7	24	34.03	-178.98	51.37	25
20071222	356	7	11	8.1	139.07	-2.41	20
20071222	356	12	26	17.47	96.81	2.09	23
20071226	360	22	4	54.67	-168.22	52.56	25
20071229	363	22	58	2.21	-168.15	52.21	15

20080101	1	18	54	59.01	146.88	-5.88	34
20080104	4	7	29	18.3	101.03	-2.78	35
20080105	5	11	1	6.11	-130.75	51.25	15
20080105	5	11	44	48.17	-130.54	51.16	10
20080106	6	5	14	20.18	22.69	37.22	75
20080109	9	14	40	0.96	-131.18	51.65	10
20080110	10	1	37	19	-127.26	43.78	13
20080115	15	17	52	15.69	-179.54	-21.98	597
20080122	22	7	55	48.88	-175.35	-15.28	6
20080122	22	10	49	21.8	-175.59	-15.42	10
20080122	22	17	14	57.95	97.44	1.01	20
20080130	30	7	32	42.8	127.69	-7.3	8
20080201	32	12	10	6.4	-179.35	-21.5	604
20080209	40	18	34	1.6	125.08	-0.24	38
20080213	44	19	58	46.13	128.64	-8.16	19
20080214	45	10	9	22.72	21.67	36.5	29
20080214	45	12	8	55.79	21.86	36.35	28
20080220	51	8	8	30.52	95.96	2.77	26
20080220	51	18	27	6	21.77	36.29	9
20080221	52	2	46	18.19	18.57	77.08	12
20080221	52	14	16	2.71	-114.87	41.15	6
20080224	55	14	46	21.47	99.93	-2.4	22
20080225	56	8	36	33.03	99.97	-2.49	25
20080225	56	18	6	3.9	99.89	-2.33	25
20080225	56	21	2	18.42	99.81	-2.24	25
20080303	63	2	37	27.12	99.82	-2.18	25
20080303	63	9	31	2.5	153.18	46.41	10
20080312	72	11	23	34.06	167.34	-16.57	13
20080312	72	11	36	55.28	167.18	-16.49	10
20080315	75	14	43	26.5	94.6	2.71	25
20080318	78	8	22	47.07	-177.44	-29.25	25
20080320	80	22	32	57.93	81.47	35.49	10
20080322	82	21	24	11.27	-178.72	52.18	132
20080329	89	17	30	50.15	95.3	2.86	20
20080409	100	11	13	17.69	168.86	-20.17	16
20080409	100	11	23	40.35	168.9	-20.18	35
20080409	100	12	46	12.72	168.89	-20.07	33
20080409	100	14	47	50.51	168.87	-20	35
20080411	102	17	45	1.94	168.84	-20.39	11
20080412	103	0	30	12.6	158.45	-55.66	16
20080415	106	22	59	51.5	-179.36	51.86	11
20080416	107	0	35	48.87	-175.7	-18.61	10
20080416	107	5	54	19.69	-179.16	51.88	13
20080418	109	20	39	7.14	-179.02	-17.34	553

20080419	110	3	12	25.18	125.69	-7.82	13
20080419	110	5	58	42.25	168.8	-20.27	14
20080419	110	10	21	12.5	125.72	-7.88	10
20080426	117	23	34	49.39	164.12	-49.09	10
20080428	119	18	33	34.2	168.95	-19.94	32
20080428	119	20	26	53.11	168.82	-20.24	35
20080502	123	1	33	37.24	-177.53	51.86	14
20080509	130	21	51	29.73	143.18	12.52	76
20080519	140	14	26	45.02	99.15	1.64	10
20080520	141	13	53	35.64	178.76	51.16	27
20080525	146	19	18	25.71	-153.78	56.09	22
20080529	150	15	46	0.32	-21.01	64	9
20080531	152	4	37	56.01	80.48	-41.2	9
20080601	153	14	31	3.01	149.66	-59.38	10
20080603	155	16	20	50.38	161.27	-10.51	84
20080603	155	22	4	27.87	120.23	-8.1	14
20080606	158	13	42	48.95	127.89	-7.49	122
20080608	160	12	25	29.71	21.52	37.96	16
20080622	174	23	56	30.03	141.28	67.7	18
20080626	178	21	19	15.58	-173.34	-20.77	38
20080627	179	11	40	13.99	91.82	11.01	17
20080628	180	12	54	46.36	91.71	10.85	15
20080703	185	3	2	37.56	-179.78	-23.37	581
20080705	187	2	12	4.48	152.89	53.88	632
20080715	197	3	26	34.7	27.86	35.8	52
20080719	201	9	27	1.46	164.49	-11.04	11
20080719	201	11	1	17.71	164.62	-11.07	10
20080719	201	22	39	52.7	-177.31	-17.34	391
20080724	206	1	43	16.14	157.58	50.97	27
20080728	210	21	40	47.36	163.1	-10.58	10
20080804	217	20	45	13.97	130.2	-5.92	173
20080810	223	8	20	33.42	91.81	11.06	20
20080819	232	16	30	13.21	-173.48	-15.09	8
20080822	235	7	47	39.58	65.39	-17.77	6
20080830	243	6	54	7.61	147.26	-6.15	75
20080901	245	4	0	39.43	-177.64	-25.39	171
20080908	252	3	3	15.93	169.11	-19.96	36
20080908	252	18	52	6.97	166.97	-13.5	110
20080910	254	11	0	34.09	55.83	26.74	12
20080911	255	0	0	2.7	127.36	1.88	96
20080929	273	15	19	31.59	-177.68	-29.76	36
20081005	279	9	12	36.07	-177.18	-30.18	10
20081005	279	15	52	49.4	73.82	39.53	27
20081005	279	22	56	28.93	69.47	33.89	10

20081019	293	5	10	33.91	-173.82	-21.86	29
20081022	296	12	55	57.39	-175.35	-18.41	233
20081023	297	10	4	35.04	145.57	-2.63	10
20081028	302	16	0	3.24	145.87	-3.49	18
20081028	302	23	9	57.65	67.35	30.64	15
20081029	303	11	32	43.13	67.46	30.6	14
20081101	306	1	13	9.64	148.71	-3.4	10
20081102	307	13	48	42.82	-174.37	51.55	36
20081104	309	18	35	45.27	168.46	-17.14	205
20081107	312	7	19	35.71	168.03	-14.83	13
20081107	312	16	4	23.35	129.07	-6.71	10
20081108	313	7	49	59	-174.23	-15.22	121
20081121	326	7	5	34.94	159.55	-8.95	118
20081122	327	16	1	1.7	101.26	-4.35	24
20081122	327	16	1	39.38	171.2	-22.52	57
20081124	329	9	2	58.76	154.32	54.2	492
20081129	334	5	59	16.58	-177.72	-18.7	386
20081206	341	10	55	26.35	124.75	-7.39	398
20081208	343	18	39	9.48	106.82	-53.01	11
20081209	344	6	23	59.75	-176.92	-31.23	18
20081209	344	17	28	58.64	168.17	-15.93	224
20081210	345	13	15	34.43	166.57	-12.34	51
20081224	359	9	11	34.29	-171.91	-17.29	9
20090103	3	19	43	50.65	132.88	-0.41	17
20090103	3	20	23	20.18	70.74	36.42	204
20090103	3	22	33	40.29	133.3	-0.69	23
20090106	6	22	48	27.25	133.43	-0.66	16
20090113	13	1	4	42.64	66.08	-13.15	10
20090115	15	7	27	20.29	170.63	-22.35	27
20090115	15	17	49	39.07	155.15	46.86	36
20090118	18	14	11	48.86	-177.95	-30.2	33
20090119	19	3	35	18.84	170.91	-22.6	12
20090119	19	3	46	37.52	170.81	-22.48	35
20090121	21	17	8	42.99	171.04	-22.69	24
20090122	22	13	40	25.99	148.51	-5.91	44
20090122	22	20	16	34.58	128.57	-7.31	146
20090217	48	3	30	53.33	-178.62	-30.72	13
20090218	49	21	53	45.16	-176.33	-27.42	25
20090306	65	10	50	29.41	-1.85	80.32	9
20090319	78	18	17	40.47	-174.66	-23.04	31
20090328	87	17	59	31.75	139.54	-2.95	93
20090330	89	7	13	7.28	-152.74	56.55	21
20090401	91	3	54	58.77	144.1	-3.52	10
20090406	96	1	32	39	13.33	42.33	8

20090407	97	4	23	33.15	151.55	46.05	31
20090415	105	20	1	34.68	100.47	-3.12	22
20090418	108	19	17	58.93	151.43	46.01	35
20090421	111	5	26	11.52	155.01	50.83	152
20090426	116	0	6	53.15	-178.58	-30.3	131
20090512	132	1	26	26.58	149.54	-5.66	89
20090516	136	0	53	51.25	-178.83	-31.55	43
20090524	144	0	58	2.11	-177.68	-31.48	4
20090602	153	2	17	3.51	167.95	-17.76	15
20090612	163	9	44	15.06	167.81	-17.61	15
20090623	174	14	19	22.35	153.78	-5.16	64
20090701	182	9	30	10.41	25.47	34.16	19
20090706	187	14	53	12.24	176.99	50.44	22
20090707	188	19	11	46.71	-72.45	75.35	19
20090715	196	9	22	29.03	166.56	-45.76	12
20090715	196	20	10	42.66	150.51	-3.38	13
20090802	214	8	54	35.55	132.9	-0.47	35
20090805	217	8	31	40.48	166.36	-45.55	10
20090810	222	4	6	31	166.09	-11.61	35
20090816	228	7	38	21.7	99.49	-1.48	20
20090818	230	21	20	47.55	-178.39	-26.06	269
20090820	232	6	35	4.43	0.94	72.2	6
20090828	240	1	51	20.4	123.43	-7.15	642
20090830	242	14	51	32.89	-172.57	-15.22	11
20090902	245	7	55	1.05	107.3	-7.78	46
20090902	245	18	0	11.41	-178.96	-29.35	275
20090907	250	16	12	22.56	110.63	-10.2	23
20090907	250	22	41	37.36	43.44	42.66	15
20090910	253	2	46	50.35	154.19	48.32	36
20090923	266	2	59	30.53	159.91	-52.96	10
20090929	272	17	48	10.99	-172.1	-15.49	18
20090929	272	17	56	5.79	-173.16	-15.35	10
20090929	272	23	45	3.46	-172.55	-15.83	10
20090930	273	10	16	9.25	99.87	-0.72	81
20091001	274	1	52	27.33	101.5	-2.52	9
20091002	275	1	7	39.26	-173.47	-16.33	8
20091002	275	15	47	9.35	174.51	-17.02	10
20091007	280	22	3	14.47	166.51	-13.01	45
20091007	280	22	18	51.24	166.38	-12.52	35
20091007	280	23	13	48.16	166.5	-13.09	31
20091008	281	2	12	39.08	166.18	-11.66	35
20091008	281	8	28	47.97	165.91	-13.3	35
20091008	281	8	34	36.96	166.49	-12.24	35
20091010	283	21	24	38.53	152.46	47.85	112

20091011	284	3	12	13.47	170.25	-22	10
20091012	285	3	15	47.28	66.69	-17.1	10
20091012	285	9	37	21.48	166.5	-12.41	42
20091013	286	5	37	23.69	-167	52.75	24
20091013	286	20	21	53.2	-167.12	52.6	14
20091014	287	18	0	21.77	-174.82	-14.91	10
20091015	288	12	11	16.41	139.54	-3.06	105
20091016	289	9	52	50.83	105.22	-6.53	38
20091019	292	22	49	38.41	-172.26	-15.36	18
20091022	295	19	51	27.52	70.95	36.52	185
20091023	296	15	14	13.06	166.05	-12.2	31
20091024	297	14	40	43.72	130.38	-6.13	130
20091025	298	7	53	52.15	-179.21	-23.09	417
20091029	302	17	44	31.23	70.72	36.39	202
20091102	306	10	47	13.24	-175.17	-24.12	9
20091105	309	9	43	20.71	160.7	-52.29	10
20091108	312	19	41	43.36	118.63	-8.21	18
20091109	313	10	44	54.77	178.34	-17.24	590
20091110	314	2	48	46.79	91.9	8.08	23
20091117	321	15	30	45.4	-131.4	52.13	3
20091122	326	7	48	20.61	-178.43	-17.79	522
20091122	326	22	47	27.48	179.47	-31.57	435
20091124	328	12	47	15.66	-174.04	-20.71	18
20091128	332	6	4	23.35	118.9	-10.41	22
20091128	332	9	21	17.98	-177.02	-29.22	28
20091209	343	9	46	3.3	170.96	-22.15	45
20091209	343	21	29	2.58	95.91	2.77	19
20091210	344	2	30	52.69	152.76	53.42	656
20091214	348	8	54	1.82	154.44	-5.96	39
20091219	353	23	19	15.55	33.82	-10.11	6
20091223	357	1	11	58.2	99.39	-1.43	19
20091226	360	8	57	27.48	131.21	-5.53	83
20100103	3	21	48	5.32	157.48	-8.74	26
20100103	3	22	36	27.96	157.35	-8.8	25
20100105	5	12	15	32.21	157.55	-9.02	15
20100105	5	13	11	42.82	157.89	-9.05	35
20100109	9	5	51	30.47	157.63	-9.13	12
20100110	10	0	27	39.32	-124.69	40.65	29
20100201	32	22	28	16.92	154.46	-6.11	32
20100205	36	6	59	5.5	99.59	-47.91	1
20100206	37	4	44	58.4	152.73	46.84	30
20100209	40	1	3	44.44	-173.49	-15.05	10
20100213	44	2	34	28.86	-174.77	-21.9	11
20100215	46	21	51	47.79	128.72	-7.22	126

20100222	53	7	0	52.91	-176.04	-23.64	25
20100304	63	14	2	27.55	167.23	-13.57	176
20100305	64	16	7	0.68	100.99	-3.76	26
20100308	67	2	32	34.71	39.99	38.86	12
20100314	73	0	57	44.7	128.13	-1.69	53
20100320	79	14	0	49.98	152.24	-3.36	414
20100405	95	10	5	46.07	125.01	-0.19	35
20100406	96	22	15	1.58	97.05	2.38	31
20100407	97	14	33	3.49	141.93	-3.77	33
20100410	100	16	54	24.25	-176.22	-20.11	273
20100411	101	9	40	25.6	161.12	-10.88	21
20100411	101	22	8	12.79	-3.54	36.97	609
20100417	107	23	15	22.02	147.29	-6.67	53
20100421	111	17	20	29.11	-173.22	-15.27	35
20100424	114	7	41	0.41	128.12	-1.91	27
20100430	120	23	11	43.34	-177.88	60.47	13
20100430	120	23	16	28.64	-177.65	60.48	14
20100505	125	16	29	3.21	101.1	-4.05	27
20100509	129	5	59	41.62	96.02	3.75	38
20100527	147	17	14	46.57	166.64	-13.7	31
20100609	160	23	23	17.35	169.49	-18.6	12
20100612	163	19	26	50.46	91.94	7.88	35
20100616	167	3	6	2.42	136.63	-2.39	13
20100616	167	3	16	27.55	136.54	-2.17	18
20100616	167	3	58	8.48	136.48	-2.33	10
20100617	168	13	6	46.59	179.72	-33.17	170
20100624	175	5	32	27.4	151.16	-5.51	40
20100626	177	5	30	19.49	161.45	-10.63	35
20100630	181	4	31	2.16	179.12	-23.31	581
20100702	183	6	4	3.13	166.49	-13.64	29
20100710	191	11	43	32.79	146	11.14	13
20100718	199	5	56	44.93	-169.85	52.88	14
20100718	199	13	4	9.41	150.43	-5.97	28
20100718	199	13	34	59.36	150.59	-5.93	35
20100718	199	19	48	4.54	-169.72	52.81	10
20100720	201	19	18	21.95	150.7	-5.91	35
20100722	203	5	3	57.09	168.17	-15.15	10
20100730	211	3	56	13.71	159.84	52.5	23
20100803	215	12	8	25.95	126.21	1.24	41
20100804	216	4	46	20.22	-177.24	-26.92	18
20100804	216	7	15	34.09	146.81	-5.5	225
20100804	216	12	58	24.2	-178.65	51.42	27
20100804	216	22	1	43.62	150.76	-5.75	44
20100810	222	5	23	44.98	168.07	-17.54	25

20100815	227	15	9	29.24	148.34	-5.69	174
20100816	228	3	30	53.31	65.65	-17.76	9
20100816	228	19	35	49	-178.83	-20.8	603
20100820	232	17	56	14.15	154.25	-6.57	19
20100821	233	5	42	52.88	96.72	2.22	24
20100903	246	11	16	6.6	-175.87	51.45	23
20100903	246	16	35	47.77	171.83	-43.52	12
20100904	247	8	52	4.1	-174	-17.37	69
20100907	250	16	13	32.22	-179.31	-15.88	10
20100908	251	11	37	31.89	169.82	-20.67	10
20100917	260	19	21	15	70.77	36.44	220
20100926	269	12	12	41.71	133.92	-5.31	30
20100929	272	17	10	51.08	133.71	-4.91	10
20100929	272	17	11	25.94	133.76	-4.96	26
20101008	281	3	26	13.71	-175.36	51.37	19
20101008	281	3	49	10.72	-175.18	51.29	27
20101025	298	14	42	22.48	100.08	-3.49	20
20101025	298	19	37	31.16	100.38	-2.95	26
20101103	307	11	18	15.57	134.07	-4.62	10
20101103	307	23	34	42.68	-174.28	-20.47	19
20101110	314	4	5	24.41	96.39	-45.46	10
20101123	327	9	1	6.89	148.97	-5.96	68
20101201	335	16	1	27.44	-178.96	-15.89	15
20101202	336	3	12	9.82	149.98	-6	33
20101213	347	1	14	42.32	155.65	-6.53	135
20101215	349	11	29	30.8	128.78	-7.26	134
20101220	354	18	41	59.63	59.17	28.44	12
20101223	357	14	0	32.7	171.18	53.13	19
20101225	359	13	16	36.9	167.9	-19.73	12
20101226	360	2	13	37.72	168.28	-19.61	13
20101228	362	8	34	17.57	-179.8	-23.41	551
20101229	363	6	54	21.01	168.21	-19.68	23
20110105	5	6	46	17.18	171.6	-22.3	135
20110109	9	10	3	42.19	168.34	-19.13	10
20110109	9	17	21	52.66	168.2	-19.22	15
20110105	5	6	46	14.63	171.63	-22.26	112
20110109	9	10	3	44.28	168.31	-19.16	24
20110109	9	17	21	51.65	168.15	-19.2	18
20110113	13	16	16	41.54	168.47	-20.63	9
20110117	17	19	20	57.21	102.65	-5.03	36
20110118	18	20	23	23.51	63.94	28.78	68
20110124	24	2	45	31.5	72.79	38.41	110
20110126	26	15	42	29.59	96.83	2.2	23
20110127	27	8	38	28.66	59.01	28.19	12

20110129	29	6	55	26.13	−6.68	70.94	6
20110131	31	6	3	27.3	−175.62	−22.01	76
20110207	38	19	53	44.16	155.18	−7.16	428
20110212	43	17	57	56.63	−175.67	−20.89	89
20110215	46	13	33	53.18	121.48	−2.5	16
20110220	51	21	43	24.15	162.12	55.92	33
20110221	52	10	57	52.41	178.39	−26.14	558
20110221	52	23	51	42.35	172.68	−43.58	5
20110307	66	0	9	36.45	160.77	−10.35	22
20110309	68	21	24	49.8	149.76	−5.99	29
20110310	69	17	8	36.86	116.72	−6.87	510
20110317	76	2	48	0.03	167.83	−17.27	17
20110326	85	22	49	41.47	−179.41	−15.85	10
20110331	90	0	11	58.3	−177.52	−16.54	15
20110401	91	13	29	10.69	26.56	35.66	59
20110403	93	14	7	9.33	−178.59	−17.64	551
20110403	93	20	6	40.39	107.69	−9.85	14
20110406	96	14	1	43.34	97.1	1.62	25
20110418	108	13	3	2.73	179.87	−34.34	86
20110423	113	4	16	54.72	161.2	−10.38	79
20110424	114	23	7	51.49	122.77	−4.59	8
20110510	130	8	55	8.93	168.23	−20.24	11
20110515	135	18	37	10.37	154.41	−6.1	40
20110605	156	11	51	12.01	146.62	−55.84	3
20110616	167	0	3	37.39	151.12	−5.96	22
20110621	172	2	4	15.91	165.55	−11.48	14
20110624	175	3	9	39.51	−171.84	52.07	52
20110626	177	12	16	38.68	136.64	−2.39	17
20110706	187	19	3	16.67	−176.22	−29.33	1
20110706	187	19	3	16.74	−176.2	−29.31	1
20110707	188	9	10	52.02	−176.73	−28.94	19

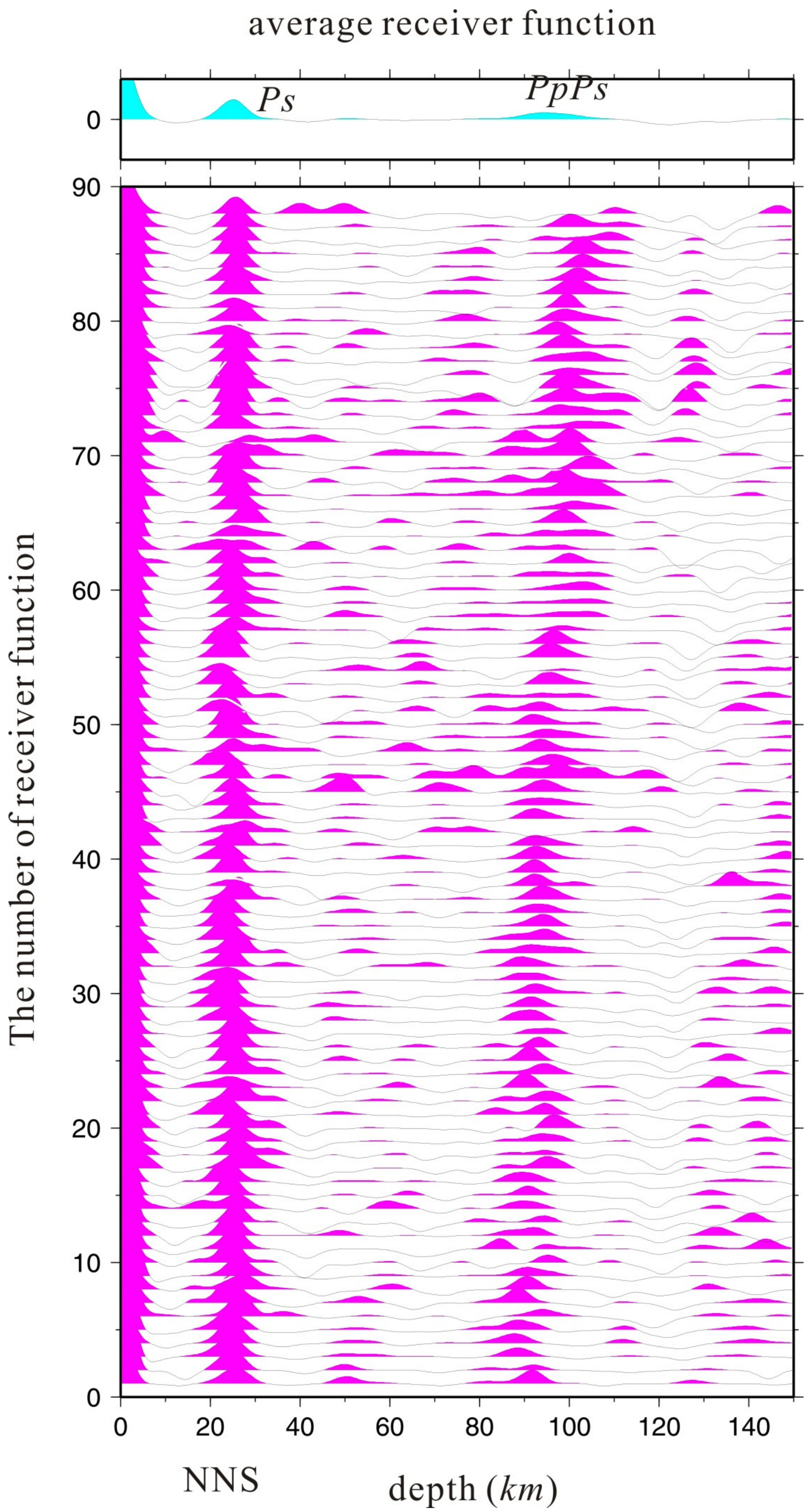

Figure S2 | **Average receiver function.** By stacking depth domain receiver functions at NNS station from different distances and directions, effects of lateral structural variation are suppressed**.**

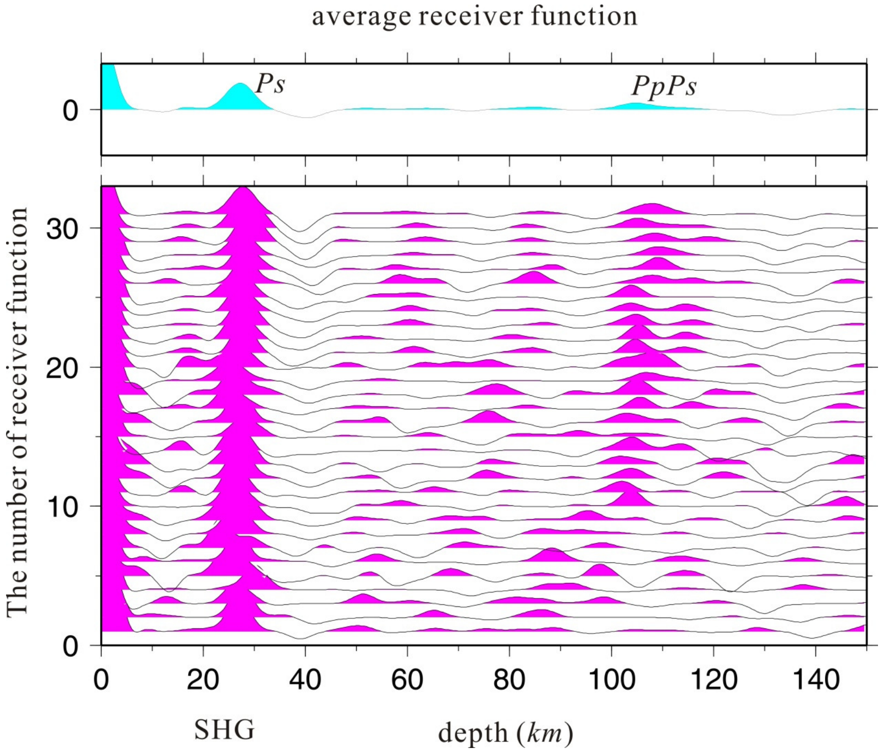

Figure S3 | **Average receiver function.** By stacking depth domain receiver functions at SHG station from different distances and directions, effects of lateral structural variation are suppressed**.**
